# The NC3Rs gateway: Accelerating scientific discoveries with new 3Rs models and technologies

**DOI:** 10.12688/f1000research.14964.1

**Published:** 2018-05-15

**Authors:** Nathalie Percie du Sert, Vicky Robinson

**Affiliations:** 1 National Centre for the Replacement, Refinement and Reduction of Animals in Research (NC3Rs), London, NW1 2BE, UK

**Keywords:** 3Rs, NC3Rs, animal research, reproducibility, animal welfare, alternative models, method development

## Abstract

This editorial introduces the NC3Rs gateway, which publishes articles and reviews on new models and technologies emerging from NC3Rs-funded research. The aim is to raise awareness about these approaches, increase confidence in their capability, and provide sufficient information to facilitate their uptake by others.

The NC3Rs is a UK-based scientific organisation that focuses on replacing, reducing and refining the use of animals in research (commonly referred to as the 3Rs). Animal research is a sensitive topic with scientific, ethical and societal challenges. Our strategy focuses on addressing the limitations of animal models, such as their translational value to humans, by funding the development of new models and technologies in a range of disciplines. We also promote best practice in animal research by tackling issues relating to animal welfare and the reproducibility of *in vivo* studies.

We are a major funder of 3Rs research and over the 14 years since the NC3Rs was established we have awarded £78 million in 3Rs R&D. While many of the 3Rs models and technologies we have funded have the potential to transform the way research is undertaken, there is often a lag between their development and their adoption into routine practice, creating a ‘3Rs valley of death’.

As an organisation committed to the highest standards in all aspects of the research we support, we want to maximise the benefits from the investment we make in projects and people, and ensure that all information arising from our awards is widely accessible. We do not want work funded by the NC3Rs to be unreported, sitting in file drawers, as this ultimately wastes animals and resources, and may skew the direction of future research.

In our experience, NC3Rs grant holders publish scientific articles detailing novel and exciting findings obtained using a new 3Rs model or technology. However, there is often a limited description of the underpinning methodology itself and its potential applications, information that is critical for the uptake of a new method, contributing to the 3Rs valley of death. The reasons for this vary, but include space limitations in journals, insufficient reward for papers that report method development alone, and a more general failure within some sectors of the biomedical research community to recognise the value of work that delivers 3Rs benefits. Launching our own publication gateway with *F1000Research* offers our grant holders an outlet to showcase their work and quickly disseminate crucial information, enabling other researchers to adopt modern approaches that bring scientific and 3Rs benefits, ultimately allowing us to tackle the 3Rs valley of death in the science we fund.

In the first instance, we will focus on publishing method and review articles, where the format has been customised to specifically highlight the 3Rs impact of the research described. This format of article is designed to showcase the 3Rs, scientific and practical benefits of the methodology, as well as its current and potential applications. Importantly, each publication will describe the technical and performance characteristics of the models and technologies, the validation process with a comparison against current ‘gold standard’ models, and a realistic assessment of the 3Rs potential. In the future, we intend to broaden the type of articles we publish, and working with *F1000Research* enables us to use innovative article formats to ensure the gateway meets the publishing needs of our grant holders. This includes the Registered Report that allows protocols to be published and reviewed before the experiment is run and the data collected, so that potential methodological issues can be addressed at a stage when they can still be corrected and encouraging researchers to share their results as soon as they become available.

Fundamentally, the NC3Rs gateway enables our grant holders to provide an honest, transparent and comprehensive account of their research, with the 3Rs impacts articulated in full. The publications will be assessed on the quality of the science rather than the novelty of the results or the direction of the findings, helping to reduce publication bias. The primary focus of the gateway is method development so our grant holders maintain the freedom to publish scientific findings in more specialist journals. Our collaboration with *F1000Research* will help to raise awareness of, and increase confidence in, new 3Rs approaches across our wide range of stakeholders, highlighting the importance and relevance of the 3Rs to biomedical research in the 21 ^st^ century.

## Data availability

No data are associated with this article.

